# Temporal change of DNA methylation subclasses between matched newly diagnosed and recurrent glioblastoma

**DOI:** 10.1007/s00401-023-02677-8

**Published:** 2024-01-20

**Authors:** Richard Drexler, Robin Khatri, Ulrich Schüller, Alicia Eckhardt, Alice Ryba, Thomas Sauvigny, Lasse Dührsen, Malte Mohme, Tammo Ricklefs, Helena Bode, Fabian Hausmann, Tobias B. Huber, Stefan Bonn, Hannah Voß, Julia E. Neumann, Dana Silverbush, Volker Hovestadt, Mario L. Suvà, Katrin Lamszus, Jens Gempt, Manfred Westphal, Dieter H. Heiland, Sonja Hänzelmann, Franz L. Ricklefs

**Affiliations:** 1 Department of Neurosurgery, University Medical Center Hamburg-Eppendorf, Martinistrasse 52, 20246 Hamburg, Germany; 2 Institute of Medical Systems Biology, University Medical Center Hamburg-Eppendorf, Hamburg, Germany; 3 Center for Biomedical AI, University Medical Center Hamburg-Eppendorf, Hamburg, Germany; 4 Institute of Neuropathology, University Medical Center Hamburg-Eppendorf, Hamburg, Germany; 5 Department of Pediatric Hematology and Oncology, Research Institute Children’s Cancer Center Hamburg, University Medical Center Hamburg-Eppendorf, Hamburg, Germany; 6 Research Institute Children’s Cancer Center Hamburg, Hamburg, Germany; 7 Department of Radiation Hematology and Oncology, University Medical Center Hamburg-Eppendorf, Hamburg, Germany; 8 III. Department of Medicine, University Medical Center Hamburg-Eppendorf, Hamburg, Germany; 9 Hamburg Center for Translational Immunology, University Medical Center Hamburg-Eppendorf, Hamburg, Germany; 10 Section of Mass Spectrometric Proteomics, University Medical Center Hamburg-Eppendorf, Hamburg, Germany; 11 Center for Molecular Neurobiology (ZMNH), University Medical Center Hamburg-Eppendorf, Hamburg, Germany; 12 Broad Institute of Harvard and MIT, Cambridge, MA USA; 13 Department of Pathology and Center for Cancer Research, Massachusetts General Hospital and Harvard Medical School, Boston, MA 02114 USA; 14 Department of Neurosurgery, Medical Center University of Freiburg, Freiburg, Germany; 15 Department of Pediatric Oncology, Dana-Farber Cancer Institute, Boston, MA USA

**Keywords:** DNA methylation, Subgroup, Glioma, Temporal, Deconvolution, Outcome

## Abstract

The longitudinal transition of phenotypes is pivotal in glioblastoma treatment resistance and DNA methylation emerged as an important tool for classifying glioblastoma phenotypes. We aimed to characterize DNA methylation subclass heterogeneity during progression and assess its clinical impact. Matched tissues from 47 glioblastoma patients were subjected to DNA methylation profiling, including CpG-site alterations, tissue and serum deconvolution, mass spectrometry, and immunoassay. Effects of clinical characteristics on temporal changes and outcomes were studied. Among 47 patients, 8 (17.0%) had non-matching classifications at recurrence. In the remaining 39 cases, 28.2% showed dominant DNA methylation subclass transitions, with 72.7% being a mesenchymal subclass. In general, glioblastomas with a subclass transition showed upregulated metabolic processes. Newly diagnosed glioblastomas with mesenchymal transition displayed increased stem cell-like states and decreased immune components at diagnosis and exhibited elevated immune signatures and cytokine levels in serum. In contrast, tissue of recurrent glioblastomas with mesenchymal transition showed increased immune components but decreased stem cell-like states. Survival analyses revealed comparable outcomes for patients with and without subclass transitions. This study demonstrates a temporal heterogeneity of DNA methylation subclasses in 28.2% of glioblastomas, not impacting patient survival. Changes in cell state composition associated with subclass transition may be crucial for recurrent glioblastoma targeted therapies.

**Supplementary Information:**

The online version contains supplementary material available at 10.1007/s00401-023-02677-8.

## Introduction

Optimal treatment of *isocitrate dehydrogenase* (*IDH*)-wildtype glioblastoma is particularly challenging due to its infiltrative nature and aggressive behavior. Current standard treatment includes maximal safe resection and adjuvant combined radiochemotherapy for newly diagnosed glioblastoma [18]. Despite this multimodality treatment, long-term local tumor control is achieved only in rare cases, and the vast majority of patients’ relapse. To date, there is no standardized treatment regimen for recurrent glioblastoma, and it is unclear which patients benefit from local or systemic therapies at this time [44]. Ringel and colleagues reported the survival benefit of resection of recurrent glioblastoma and established recurrent surgery as an option for second-line therapy when this can be done safely [39]. Additional studies confirmed the prolonged survival after recurrent surgery [3, 50, 62]. Nevertheless, a major challenge in the search for a more effective surgical and therapeutic regimen is the extensive inter- and intratumoral heterogeneity, which is considered one of the main factors for treatment failure in glioblastoma [36, 37].

Genome-wide DNA methylation profiling has recently emerged as a tool enabling accurate molecular classification of central nervous system (CNS) tumors and has the potential to further stratify patients according to their molecular pathological characteristics [6, 7]. It allows the subdivision of glioblastoma into different methylation subclasses such as the most abundant *receptor tyrosine kinase I* (*RTK I*), *receptor tyrosine kinase II* (*RTK II*), and mesenchymal (MES) subclasses [49]. Recently, methylation-based classification of glioblastoma has become increasingly important for predicting therapeutic response and aids in clinical decision-making for distinct subclasses [11–13, 38, 60]. One issue that needs to be addressed when generally classifying glioblastoma by subgroups is heterogeneity within the tumor itself [23, 34]. In addition, the extent of heterogeneity appears to influence patient prognosis [23, 34]. Focusing on DNA methylation subclasses of glioblastoma, spatial heterogeneity of these subclasses has been analyzed in newly diagnosed glioblastoma, with studies reporting varying degrees of heterogeneity [53, 59].

Drawing on these findings, an important consideration is whether temporal changes in DNA methylation subclasses occur during glioblastoma progression, what factors contribute to a potential subclass transition, and the extent to which such a transition influences patient outcome. To explore this, we conducted global DNA methylation profiling in 47 patients, comparing tissue and serum samples from the initial and recurrent surgeries.

## Methods

### Study population

Matched tissue samples from 47 patients diagnosed with *IDH*-wildtype glioblastoma, who underwent initial and recurrent surgeries at University Medical Center Hamburg-Eppendorf and University Medical Center Freiburg (both Germany), were analyzed. Diagnosis was based on the current WHO classification [29]. The extent of resection (EOR) was stratified into gross total resection (GTR), near-GTR, and partial resection (PR). A GTR was defined as a complete removal of contrast-enhancing parts, a near-GTR as a removal of more than 90% of the contrast-enhancing parts, whereas a resection of lower than 90% was defined as PR/biopsy. The EOR of contrast-enhancing parts was evaluated by magnetic resonance imaging (MRI) performed within to 48 h after index surgery. Overall survival (OS) was calculated from diagnosis until death or last follow-up, and progression-free survival (PFS) from diagnosis until progression according to Response Assessment in Neuro-Oncology (RANO) criteria based on local assessment [58]. The progression-to-progression survival (PPS) was calculated from recurrence surgery until next progression, and progression-to-overall survival (POS) from recurrence surgery until death or last follow-up.

### DNA methylation profiling

DNA was extracted from tumor tissue and bulk plasma and analyzed for genome-wide DNA methylation patterns using the Illumina EPIC (850 k) array. The tumor tissue of interest for performing DNA methylation was chosen by a board-certified neuropathologist of the Department of Neuropathology, University Medical Center Hamburg-Eppendorf, Germany. Processing of DNA methylation data was performed with custom approaches [7].

### Inclusion criteria

Methylation profiling results from first and recurrent surgery were submitted to the molecular neuropathology (MNP) methylation classifier v12.8 hosted by the German Cancer Research Center (DKFZ) [6]. Patients were included if the calibrated score for methylation class family glioblastoma, *IDH*-wildtype was > 0.84 at time of diagnosis [7]. In addition, patients with a score below 0.84 but above 0.7 with a combined gain of chromosome 7 and loss of chromosome 10 or amplification of epidermal growth factor receptor (*EGFR*) were included in accordance with cIMPACT criteria [5]. Furthermore, a class member score of ≥ 0.5 for one of the glioblastoma subclasses was required. In addition, the following clinical criteria were defined: age above 18 years at time of diagnosis, local tumor progression at first recurrence, and availability of data for OS and PFS.

### t-SNE embeddings

To compute t-SNE embeddings, first, 50 principal components (PCs) were computed for 25,000 of the most variable CpG sites. Subsequently, the class TSNE() from the Python package scikit-learn (v1.2.2) was fitted on the PCs with the perplexity argument set to 10, and was used to transform the PCs into t-SNE embeddings. The t-SNE results were visualized using the scatter function from the Python package Matplotlib (v3.8.2).

### Differentially methylated probes and gene set enrichment

Differentially methylated probes in newly diagnosed glioblastoma with subclass transition were computed using the function dmpfinder from the R package minfi (v1.40.0). The resulting data frame was filtered with a *p* value cutoff of 0.01 and an absolute beta-value difference of 0.1. This led to the identification of 1962 differentially methylated probes (1415 hypermethylated probes and 547 hypomethylated probes). Gene annotations for these CpGs were extracted from the Illumina EPIC Manifest file (v1.0 B5). Only the following gene region feature categories were retained: TSS200, TSS1500, 1stExon, and 5’UTR. The resulting 493 hypermethylated and 113 hypomethylated genes were then used for gene set enrichment analysis based on GO biological processes (2023) with the R Package clusterProfiler (v4.2.0).

### Copy number alterations

Genome-wide DNA methylation profiling was further used to analyze chromosomal copy number alterations and to provide information regarding gene amplifications, gains and losses as routinely done by the tumor classifier of the DKFZ. Genes and chromosomal regions were manually evaluated for differences in copy numbers from baseline and compared with other indicator genes on the array. For the assessment of relevant deviations, we followed the recommendations described by Capper et al. [7].

### Cell state composition analysis

To infer the abundance of cell type and cell state in the samples, we applied the Silverbush et al. deconvolution method [47] to each sample that underwent bulk DNA methylation analysis using EPIC V2.0 arrays. The deconvolution method is a reference free method that uses a hierarchical matrix factorization approach inferring both cell types and the cell states therein. The method was trained on the DKFZ GBM cohort and tested on the TCGA GBM cohort and was shown to be able to infer the abundance of cell types in the microenvironment (immune cells, glia, and neurons) as well as malignant cell states (malignant stem-like cells component and two differentiated cells components). The stem-like component provides a deconvoluted estimation of the fraction of stem-like cells in a glioblastoma sample, specifically those exhibiting a transcriptional cell state of OPC-like or NPC-like [32]. Both Differentiated 1 and Differentiated 2 represent the estimated fraction of differentiated cells in a glioblastoma sample, particularly those with a transcriptional cell state of MES or AC-like [32]. We applied the method as described in Silverbush et al. using the cell type and cell state encoding provided in the manuscript and via the engine provided in EpiDISH [63] package, with Robust Partial Correlations (RPC) method and maximum iterations of 2000.

### Cell type deconvolution

#### Processing of methylation arrays

All Idats corresponding to methylation array data of tumor tissue and patients serum were processed similarly using the minfi package in R (version 1.40.0). The data was processed using the preprocessIllumina function. Only probes with detection *p* values < 0.01 were kept for further analysis. Also, probes with < 3 beads in at least 5% of samples, as well as all non-CpG probes, SNP-related probes, and probes located on X and Y chromosomes were discarded. The CpG intensities were converted into beta values representing total methylation levels (between 0 and 1).

#### Cell type deconvolution

Cell type deconvolution was applied to methylation arrays of tumor tissue and serum. Non-negative least square (NNLS) linear regression was used in deconvolving the beta values of methylation arrays into cell type components [30, 41, 51]. As a reference, a publicly available signature was obtained from Moss et al. (2018) consisting of gene expressions for 25 cell type components (Monocytes_EPIC, B-cells_EPIC, CD4T-cells_EPIC, NK-cells_EPIC, CD8T-cells_EPIC, Neutrophils_EPIC, Erythrocyte_progenitors, Adipocytes, Cortical_neurons, Hepatocytes, Lung_cells, Pancreatic_beta_cells, Pancreatic_acinar_cells, Pancreatic_duct_cells, Vascular_endothelial_cells, Colon_epithelial_cells, Left_atrium, Bladder, Breast, Head_and_neck_larynx, Kidney, Prostate, Thyroid, Upper_GI, Uterus_cervix) and 6,105 unique CpGs[30].

### Proteomics

#### Proteomic processing of human glioblastoma samples

Formalin-fixed paraffin embedded (FFPE) samples of tumors were obtained from tissue archives from the neuropathology unit in Hamburg. Tumor samples were fixed, dehydrated, embedded in paraffin, and sectioned at 10 μm for microdissection using standard laboratory protocols. For paraffin removal FFPE tissue sections were incubated in 0.5 mL n-heptane at room temperature for 30 min, using a ThermoMixer (ThermoMixer^®^ 5436, Eppendorf). Samples were centrifuged at 14.000 g for 5 min and the supernatant was discarded. Samples were reconditioned with 70% ethanol and centrifuged at 14.000 g for 5 min. The supernatant was discarded. The procedure was repeated twice. Pellets were dissolved in 150 µL 1% w/v sodium deoxycholate (SDC) in 0.1 M triethylammonium bicarbonate buffer (TEAB) and incubated for 1 h at 95 °C for reverse formalin fixation. Samples were sonicated for 5 s at an energy of 25% to destroy interfering DNA. A bicinchoninic acid (BCA) assay was performed (Pierce^™^ BCA Protein Assay Kit, Thermo Scientific) to determine the protein concentration, following the manufacturer’s instructions. Tryptic digestion was performed for 20 µg protein, using the Single-pot, solid-phase-enhanced sample preparation (SP3) protocol, as described by Hughes et al. [20].  Eluted Peptides were dried in a Savant SpeedVac Vacumconcentrator (Thermo Fisher Scientific, Waltham, USA) and stored at -20° until further use. Directly prior to measurement dried peptides were resolved in 0.1% FA to a final concentration of 1 μg/μl. In total 1 μg was subjected to mass spectrometric analysis.

#### Liquid chromatography–tandem mass spectrometer parameters

Liquid chromatography–tandem mass spectrometer (LC–MS/MS) measurements were performed on a quadrupole-ion-trap-orbitrap mass spectrometer (MS, QExactive, Thermo Fisher Scientific, Waltham, MA, USA) coupled to a nano-UPLC (Dionex Ultimate 3000 UPLC system, Thermo Fisher Scientific, Waltham, MA, USA). Tryptic peptides were injected to the LC system via an autosampler, purified and desalted by using a reversed phase trapping column (Acclaim PepMap 100 C18 trap; 100 μm × 2 cm, 100 A pore size, 5 μm particle size; Thermo Fisher Scientific, Waltham, MA, USA), and thereafter separated with a reversed phase column (Acclaim PepMap 100 C18; 75 μm × 25 cm, 100 A pore size, 2 μm particle size, Thermo Fisher Scientific, Waltham, MA, USA). Trapping was performed for 5 min at a flow rate of 5 µL/min with 98% solvent A (0.1% FA) and 2% solvent B (0.1% FA in ACN). Separation and elution of peptides were achieved by a linear gradient from 2 to 30% solvent B in 65 min at a flow rate of 0.3 µL/min. Eluting peptides were ionized by using a nano-electrospray ionization source (nano-ESI) with a spray voltage of 1800 V, transferred into the MS, and analyzed in data dependent acquisition (DDA) mode. For each MS1 scan, ions were accumulated for a maximum of 240 ms or until a charge density of 1 × 1^6^ ions (AGC target) were reached. Fourier-transformation-based mass analysis of the data from the orbitrap mass analyzer was performed by covering a mass range of 400–1200 m/*z* with a resolution of 70,000 at *m*/*z* = 200. Peptides with charge states between 2 + –5 + above an intensity threshold of 5,000 were isolated within a 2.0* m*/*z* isolation window in top-speed mode for 3 s from each precursor scan and fragmented with a normalized collision energy of 25%, using higher energy collisional dissociation (HCD). MS2 scanning was performed, using an orbitrap mass analyzer, with a starting mass of 100 m/z at an orbitrap resolution of 17,500 at *m*/*z* = 200 and accumulated for 50 ms or to an AGC target of 1 × 105. Already fragmented peptides were excluded for 20 s.

#### Raw data processing

Proteomics samples were measured with liquid chromatography tandem mass spectrometry (LC–MS/MS) systems and processed with Proteome Discoverer 3.0 and searched against a reviewed FASTA database (UniProtKB: Swiss-Prot, Homo sapiens, February 2022, 20,300 entries). To cope with protein injection amount differences, the protein abundances were normalized at the peptide level. Perseus 2.0.3 was used to obtain log2 transformed intensities. The imputation was performed using the Random Forest imputation algorithm (Hyperparameters: 1000 Trees and 10 repetitions) in R, version 4.3.

#### Differential protein abundance

The protein abundances of the nine samples were corrected for the batch effect using ComBat [24]. Protein level normalization was performed by centering protein abundances per file around the median value. For cases, measured multiple times, the mean value of abundances across all measurements was calculated. Values are log2 normalized (Peptide level), RF-imputed, batch-effect corrected normalized (Protein level) protein abundance (Mean value per case, across all measurements). For differential abundance analysis, *p* values and log2 fold changes for each quantified protein (total 4716) were estimated using the empirical Bayes statistical test as implemented in the limma R package [35].

#### Weighted correlation network analysis (WGCNA)

The WGCNA package in R (version 1.70.3) was used to identify protein co-expression modules [28]. The minimum module size was set to 15 and a merging threshold of 0.40 was defined. Based on the assessment of scale-free topology, soft-power of 18 was selected. To construct modules, first we corrected for any technical batch effect using empirical Bayes-moderated adjustment using empiricalBayesLM function of WGCNA. Modules were assessed based on their correlation with traits (No transition and transition) and their levels of significance (associated with two-tailed Student’s *t* test). The significant modules (*p* < 0.05) were used for further analysis. All genesets within a module were used for overrepresentation analysis using clusterProfiler package in R (Version 4.2.0) [61]. Further, to identify cell type enrichment within each module, enrichr API in python was used (Package maayanlab_bioinformatics, version 0.5.4 with PanglaoDB library available within the package) [40]. To assess the module scores on single cells, Scanpy’s score_genes function was used to calculate module scores using core glioblastoma single-cell atlas [27].

#### Detection of soluble factors in patient serum

Plasma from glioblastoma patients was taken 1 h prior primary surgery and then isolated by double spin centrifugation of whole blood. Samples were aliquoted and stored at − 80 °C before use. Soluble factors were detected using the LEGENDplex Neuroinflammation Panel 1 (Biolegend, San Diego, CA, USA) according to the supplier’s protocol were simultaneously determined using a multiplex bead-based immunoassay (LEGENDPlexTM Human Neuroinflammation Panel 1, Cat. No. 740795, Biolegend, USA). Data were acquired using the BD LSR Fortessa and Beckman Coulter Cytoflex LX flow cytometer and analyzed with the BioLegend LEGENDplex software.

#### DNA tumor purity

Tumor-purity was calculated using the RF_purify Package in R [22]. This package uses the “absolute” method which measures the frequency of somatic mutations within the tumor sample and relates this to the entire DNA quantity [8].

#### 3D volumetric segmentation

We analyzed T1-weighted as well as T2-weighted FLAIR (Fluid attenuated inversion recovery) from MRI using the program BRAINLAB. To measure tumor volume, the tumor region of interest was delineated with the tool “Smart Brush” enabling a multiplanar 3D reconstruction. Volume of contrast enhancement and FLAIR hyperintensity was assessed in cm^3^.

#### Ethics statement

This study was approved by the medical ethics committee of the Hamburg chamber of physicians (PV4904). Informed written consent was obtained from all patients.

### Statistical analysis

Gaussian distribution was confirmed by the Shapiro–Wilk normality test. For parametric data, unpaired two-tailed Student’s *t* test or one-way ANOVA with Tukey’s post hoc tests to examine pairwise differences were used as indicated. Survival curves were visualized as results from the Kaplan–Meier method applying two-tailed log rank analyses for analyzing statistical significance. In general, a *p* value less than 0.05 was considered statistically significant for all experiments. Statistical analyses were performed using SPSS Inc. (Version 29, Chicago, IL, USA). Data illustrations were performed using GraphPad Prism 10. Alluvial plots were graphed with R.

## Results

### Study population

In this study, we analyzed a cohort of 47 patients who underwent surgery for newly diagnosed glioblastoma as well as tumor recurrence, with their tumors subjected to global DNA methylation profiling. Clinical data were available for 32 patients, among whom 11 (34.4%) were female and 21 (65.6%) were male, with a mean age at the time of the initial surgery of 59.4 ± 11.5 years (Table 1). All 32 tumors exhibited contrast enhancement on preoperative MRI and were localized supratentorially, with 20 (62.5%) in eloquent areas. Gross total resection (GTR) was achieved in 62.5%, near-GTR in 28.1%, and partial resection in 9.4% of patients (Table 1).

**Table 1:** Clinical and methylation characteristics of the study population at time of first and recurrence surgery

Feature	*N* = 32	No subclass transition (*n* = 22)	Subclass transition (*n* = 10)	*p* value
First resection				
Age at 1st surgery, mean (SD)	59.4 (11.5)	59.1 (12.4)	59.9 (9.9)	0.87
Gender, *n* (%)				
Female	11 (34.4)	8 (36.4)	3 (30.0)	0.73
Male	21 (65.6)	14 (63.6)	7 (70.0)	
Preoperative seizures, *n* (%)	15 (46.9)	10 (45.5)	5 (50.0)	0.81
Location, *n* (%)				
Frontal	8 (25.0)	5 (22.7)	3 (30.0)	0.66
Parietal	17 (53.1)	11 (50.0)	6 (60.0)	0.59
Temporal	15 (46.9)	8 (36.4)	7 (70.0)	0.08
Occipital	3 (9.4)	2 (9.1)	1 (10.0)	0.94
Eloquent	20 (62.5)	14 (63.6)	6 (60.0)	0.84
Side, *n* (%)				
Left	19 (59.4)	14 (63.6)	5 (50.0)	0.47
Right	13 (40.6)	8 (36.4)	5 (50.0)	
Preoperative CE volume, [cm^3^], mean (SD)	20.5 (22.2)	13.8 (20.6)	29.5 (25.1)	0.40
Preoperative FLAIR volume, [cm^3^], mean (SD)	53.9 (45.9)	33.1 (30.3)	81.8 (53.9)	0.18
Extent of 1st resection, *n* (%)				
GTR	20 (62.5)	17 (77.3)	3 (30.0)	**0.04**
Near GTR	9 (28.1)	4 (18.2)	5 (50.0)	
Partial resection	3 (9.4)	1 (4.5)	2 (20.0)	
Karnofsky prior 1st line adjuvant therapy, [%], mean (SD)				
1st line adjuvant therapy, *n* (%)				
None	4 (12.5)	3 (13.6)	1 (10.0)	0.77
Combined radiochemotherapy	28 (87.5)	19 (86.4)	9 (90.0)	
Radiation dosage, [Gy], mean (SD)	59.6 (2.9)	60.2 (0.9)	58.2 (4.9)	0.09
No. of cycles of Temozolomide, mean (%)	3.8 (2.4)	3.9 (2.2)	3.4 (2.9)	0.59
Max. TMZ dosage, [mg/m^2^], mean (SD)	159.0 (31.4)	159.2 (30.3)	158.2 (37.7)	0.95
Time 1st surgery to adjuvant treatment start, [days], mean (SD)	27.8 (9.7)	28.9 (10.1)	25.1 (8.8)	0.37
Time end combined treatment to start 1st cycle TMZ, [days], mean (SD)	23.4 (12.4)	23.7 (13.9)	22.0 (4.0)	0.79
Time 1st to 2nd surgery, [months], mean (SD)	15.6 (16.7)	15.4 (17.6)	16.1 (15.3)	0.91
Absolute DNA tumor purity, mean (SD)	0.54 (0.09)	0.55 (0.09)	0.52 (0.11)	0.46
Estimate DNA tumor purity, mean (SD)	0.80 (0.07)	0.82 (0.06)	0.78 (0.08)	0.20
Re-resection				
Extent of 2nd resection, *n* (%)				
GTR	12 (37.5)	11 (50.0)	1 (10.0)	0.09
Near GTR	15 (46.9)	8 (36.4)	7 (70.0)	
Partial resection	5 (15.6)	3 (13.6)	2 (20.0)	
Karnofsky prior 2nd line adjuvant therapy, mean (SD)	78.4 (14.2)	80.5 (13.9)	74.0 (14.3)	0.24
2nd line adjuvant therapy, *n* (%)				
None	2 (6.3)	2 (9.1)	0 (0.0)	**0.04**
Stupp	11 (34.4)	7 (31.8)	4 (40.0)	
Radiation + procarbazin/CCNU	7 (21.9)	7 (31.8)	0 (0.0)	
Temzolomide	2 (6.3)	0 (0.0)	2 (20.0)	
Procarbazin/CCNU	3 (9.4)	3 (13.6)	0 (0.0)	
Re-radiation	1 (3.1)	0 (0.0)	1 (10.0)	
Experimental	6 (18.8)	3 (13.6)	3 (30.0)	
DNA methylation profiling				
Subclass 1st surgery, *n* (%)				
RTK I	8 (25.0)	5 (22.7)	3 (30.0)	0.77
RTK II	15 (46.9)	10 (45.5)	5 (50.0)	
MES	9 (28.1)	7 (31.8)	2 (20.0)	
Calibrated score 1st surgery, mean (SD)	0.95 (0.09)	0.94 (0.09)	0.96 (0.07)	0.57
Family member score 1st surgery, mean (SD)	0.72 (0.18)	0.74 (0.19)	0.70 (0.16)	0.57
Subclass 2nd surgery, *n* (%)				
RTK I	6 (18.8)	5 (22.7)	1 (10.0)	0.13
RTK II	12 (37.5)	10 (45.5)	2 (20.0)	
MES	14 (43.8)	7 (31.8)	7 (70.0)	
Calibrated score 2nd surgery, mean (SD)	0.95 (0.09)	0.95 (0.08)	0.95 (0.11)	0.93
Family member score 2nd surgery, mean (SD)	0.73 (0.16)	0.77 (0.16)	0.66 (0.16)	0.11

*P* values in bold refer to a significant value below 0.05

The majority (87.5%) received radiochemotherapy as adjuvant treatment between the initial and recurrent surgeries. The median time between the initial and recurrent surgeries was 15.6 ± 16.7 months. At recurrence surgery, GTR or near-GTR was achieved in 27 (84.4%) patients, and partial resection was performed in 5 (15.6%) patients. Following recurrence surgery, 2 (6.3%) patients did not receive adjuvant treatment due to a low Karnofsky performance score (Table 1). 

### Mesenchymal transition is most frequent in recurrent glioblastoma

**Fig. 1:**
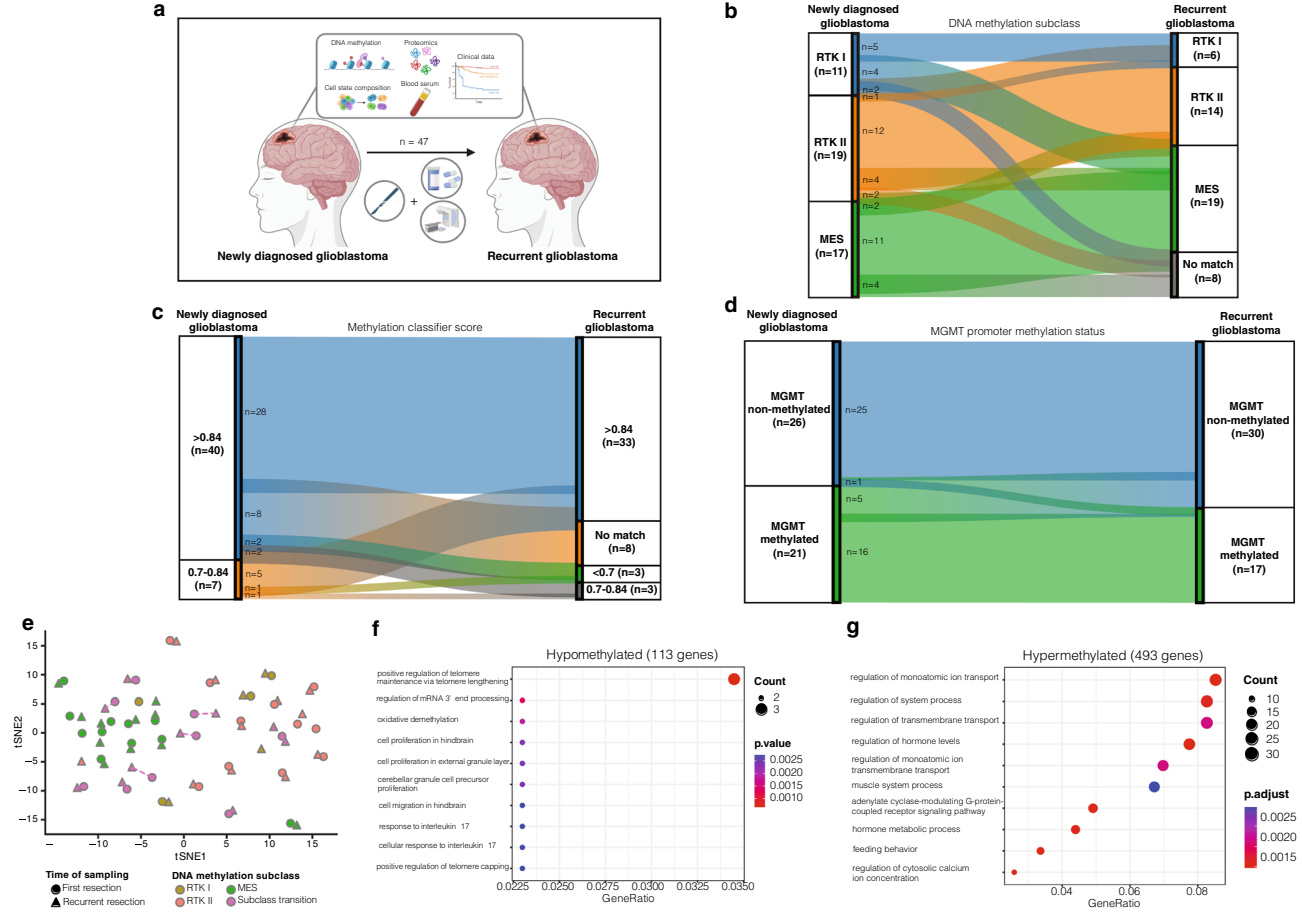
Analysis of DNA methylation profiling between tumor tissue obtained from first and recurrent resection of *IDH*-wildtype glioblastoma. **a** Study workflow. **b**–**d** Sankey plots showing a possible transition of **b** DNA methylation subclasses, **c** calibrated scores, and **d** *MGMT* promoter methylation status. **e** T-distributed stochastic neighbor embedding shows methylation subclasses *RTK I* (gold), *RTK II* (red), and MES (green) from first resection, and tumors with a subclass transition (purple) at time of recurrence. **f** Gene set enrichment analysis of differentially methylated CpG sites in hypomethylated genes of newly diagnosed glioblastomas undergoing subclass transition compared to glioblastomas without transitions. **g** Gene set enrichment analysis of differentially methylated CpG sites in hypermethylated genes of newly diagnosed glioblastomas undergoing subclass transition compared to glioblastomas without transitions. *RTK* receptor tyrosine kinase, *MES* mesenchymal

After applying DNA methylation profiling with the DKFZ methylation classifier, patients were stratified based on their methylation subclass, with *RTK II* being the most common subclass at the time of diagnosis (40.4%, Fig. 1b). Eight (17.0%) tumors had a classifier result designated as “no match” by the time of recurrence (Fig. 1b). DNA purity and input amount were checked to ensure sufficient quality of these samples (Supplementary Fig. 1a, b). Among the remaining 39 (83.0%) recurrent glioblastomas, the majority (48.7%) were of the MES subclass (Fig. 1b). Overall, a change in the dominant DNA methylation subclass was observed in 11 of 39 (28.2%) glioblastomas that had a valid classifier output at both time points (Fig. 1b). In these tumors, most transitions (72.7%) were to the MES subclass (Fig. 1b). Calibrated scores for “IDH-wildtype glioblastoma” and family member scores for methylation subclass were comparable between matched tumor tissues (Table 1, Fig. 1c). A change in the *MGMT* promoter methylation status was observed in 6 (12.8%) patients (Fig. 1d).

We analyzed clinical, surgical, and treatment-related factors potentially influencing the temporal transition of the DNA methylation subclass (Table 1). From this analysis, transition was more likely after incomplete removal of contrast-enhanced tumor parts (*p* = 0.04, Table 1). Treatment-related factors such as radiation dose (*p* = 0.09), maximum temozolomide dose (*p* = 0.95), number of TMZ cycles (*p* = 0.59), and time intervals between surgery and treatment initiation (*p* = 0.37) or between the first surgery and recurrence surgery (*p* = 0.91) did not correlate with subclass transition (Table 1).

### Deconvolution reveals an increased stem cell-like state but decreased proportion of immune cells in glioblastomas with mesenchymal transition at the time of diagnosis

For a deeper insight into the epigenetic mechanisms underlying glioblastoma with a subclass change upon recurrence, we explored the distinctions in methylation signatures (Fig. 1e). To address this, we conducted a differential methylation analysis, focusing on significantly differentially methylated CpG sites in both hypomethylated (Fig. 1f) and hypermethylated (Fig. 1g) genes between newly diagnosed glioblastomas that did not undergo a subclass transition and those that did.

**Fig. 2:**
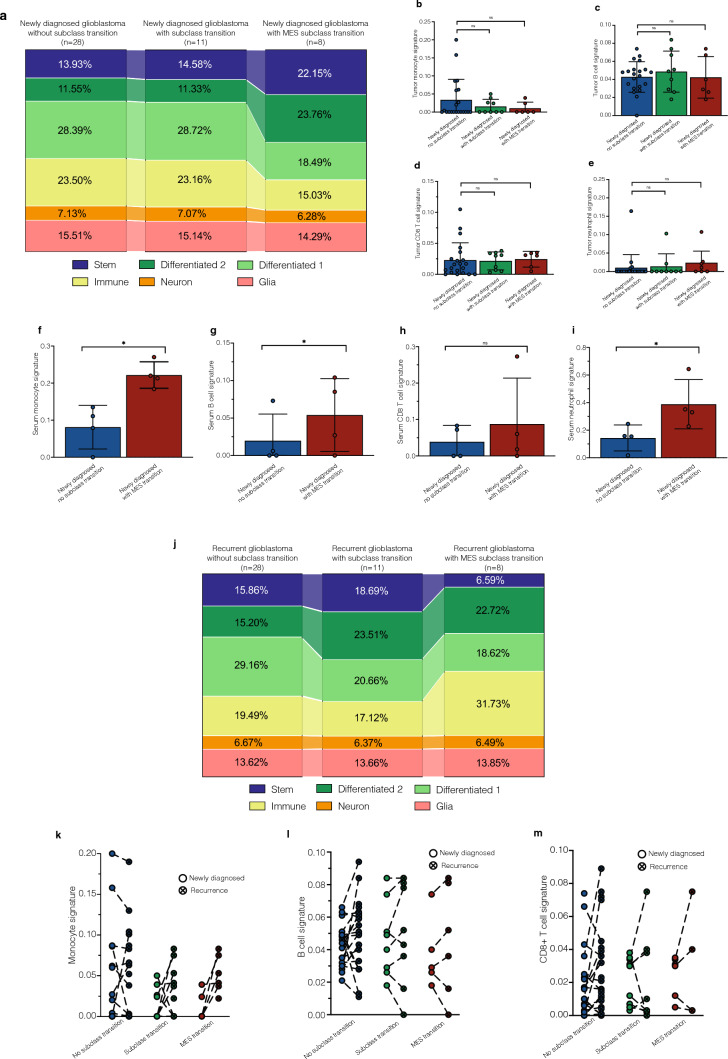
Cell state composition analysis from tissue and serum. **a** Cell state composition analysis of newly diagnosed glioblastoma separated to a potential subclass transition. **b**–**e** Immune cell signatures calculated from tumor tissue of first surgery. **f**–**i** Signatures of circulating serum levels of immune cells in patients with newly diagnosed *IDH*-wildtype glioblastoma. **j** Cell state composition analysis of recurrent glioblastoma tissue separated to a potential subclass transition. **k**–**m** Soluble factors with significantly different serum levels at time of diagnosis between glioblastoma with and without mesenchymal transition. *MES* mesenchymal

Given the identification of distinct cell populations within DNA methylation subclasses in prior studies [48], and the alterations in cell composition during glioblastoma recurrence [52], we investigated whether specific cell states at the time of diagnosis were linked to subsequent subclass transitions. To explore this hypothesis, we employed a methylation-based deconvolution method, integrating bulk and cell-type-specific tumor datasets to analyze cell state compositions [47]. There were no significant variations in cell composition observed between newly diagnosed glioblastoma cases without subclass transition and those with subclass transition (Fig. 2a). However, within the subgroup undergoing a subsequent mesenchymal transition, there was a notable increase in the presence of malignant stem cell-like and differentiated 2-like states at the time of diagnosis (Fig. 2a). Furthermore, this subgroup with a mesenchymal transition exhibited a decreased proportion of immune cells compared to newly diagnosed tumors without a subclass transition (Fig. 2a). A more detailed breakdown of the immune compartment into distinct immune cell types revealed no significant differences between glioblastomas with or without mesenchymal transition (Fig. 2b–e). Interestingly, an increase in circulating monocyte, B-cell and neutrophil signatures, inferred from 850 k arrays, was observed in the serum at the time of diagnosis in a subset of patients with mesenchymal transition (Fig. 2f–i).

Analyzing the cell compositions in tumor tissue at the time of recurrence showed no significant differences in cell states for tumors with or without subclass transition (Fig. 2j). Intriguingly, tumors with a mesenchymal transition demonstrated a highly elevated immune component but a decreased stem-like state, resulting in an opposite cell composition compared to the initial diagnosis (Fig. 2j). In addition, further analyses revealed an increased signature of monocytes, B cells, and CD8 + T cells in the tumor tissue of recurrent glioblastomas with mesenchymal transition (Fig. 2k–m).

### TREM-2, IL-6, and IL-18 in serum change significantly during mesenchymal transition

**Fig. 3:**
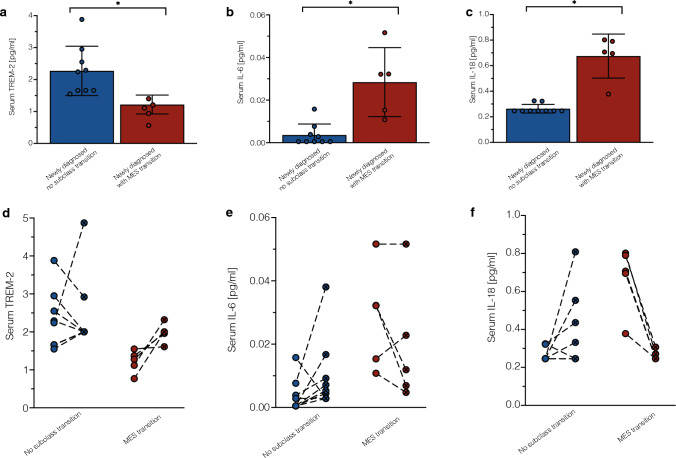
Soluble factor analysis of patients’ serum at time of diagnosis and recurrence. **a**–**c** Serum levels of soluble factors TREM-2, IL-6, and IL-18 at time of diagnosis between tumors with a mesenchymal transition and without a subclass transition. **d**–**f** Comparison of serum levels of soluble factors TREM-2, IL-6, and IL-18 between first (blank dot) and recurrent (x-shaped dot) surgery with respect to a potential mesenchymal transition. *MES* mesenchymal, *TREM-2* triggering receptor expressed on myeloid cells, *IL* interleukin

Since we demonstrated a lower immune cell compartment at time of diagnosis in tumor tissue with a mesenchymal subclass transition which is significantly increased in recurrent tissue, we pondered the question if we could identify a soluble factor as potential biomarker for a subclass transition. To address, we performed a bead-based immunoassay using LEGENDplex and quantified various soluble analytes of a neuroinflammation panel (see methods). This analysis was possible for 14 matched serum pairs of newly diagnosed and recurrent glioblastoma, of which 5 glioblastomas experienced a mesenchymal transition (Fig. 3). Among all soluble factors in the panel, *triggering receptor expressed on myeloid cells (TREM)-2, Interleukin (IL)-6*, and *IL-18* showed significant changes between the two subgroups at first diagnosis (Fig. 3a–c). While IL-6 (*p* < 0.01) and IL-18 (*p* < 0.01) serum levels were significantly increased in serum of newly diagnosed tumors with subsequent mesenchymal transition, TREM-2 (*p* = 0.01) levels were decreased (Fig. 3a–c). However, the aforementioned cytokines IL-6 and IL-18 had lower serum levels at time of recurrence of mesenchymal transitioned tumors (Fig. 3d–f). In summary, glioblastoma cases exhibiting a mesenchymal transition demonstrate elevated levels of stem cell-like states within the tumor tissue, while the immune compartment shows a decrease. Interestingly, there is a concurrent increase in immune cell signature observed in the serum of these patients at the time of diagnosis.

### Tumor proteome shows altered metabolism and an enrichment of AC-like cells in transitioning tumors at time of diagnosis

**Fig. 4:**
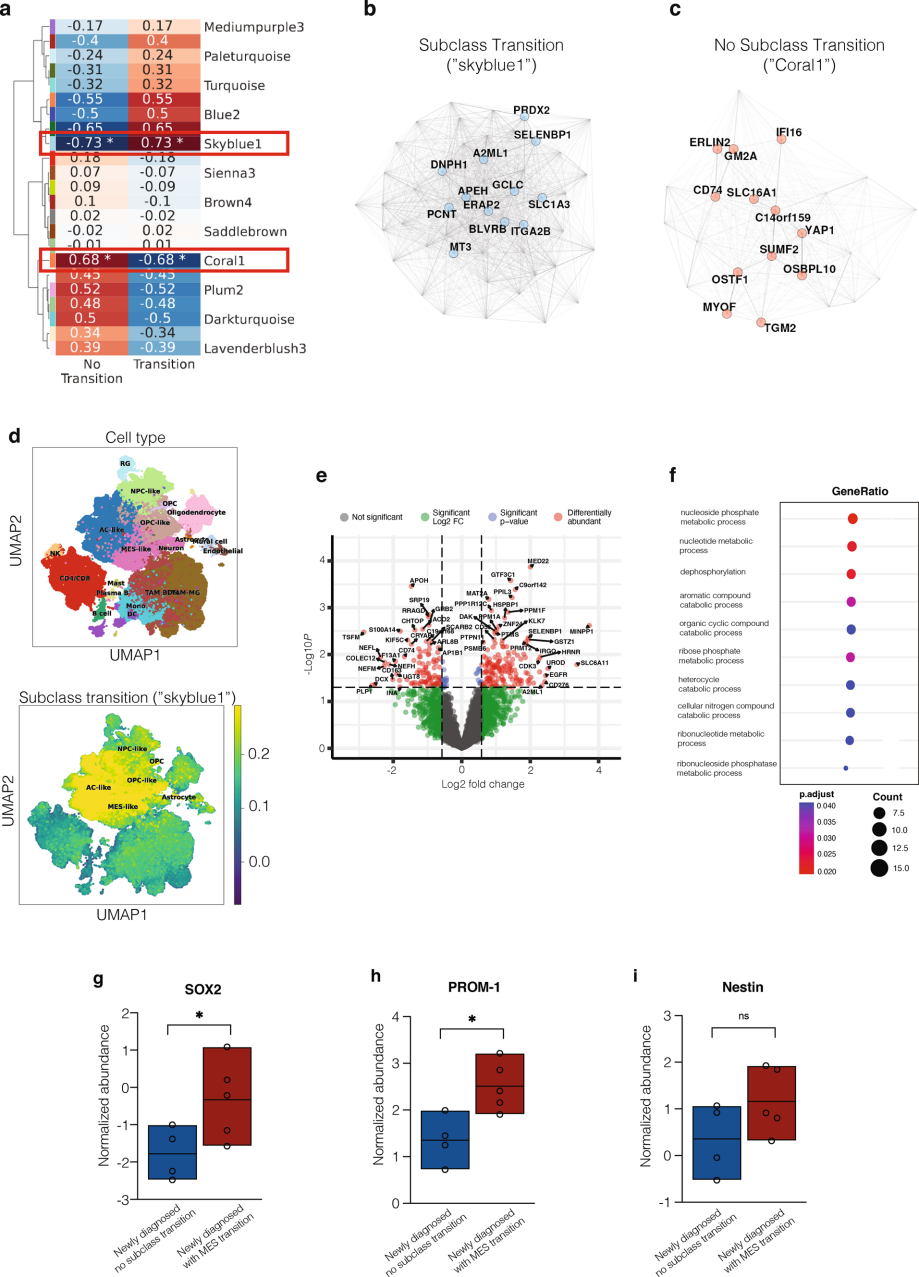
Proteomic profiling of newly diagnosed *IDH*-wildtype glioblastomas with and without a subclass transition. **a** WCGNA analysis showed differentially correlated proteome modules between both subgroups. Tumors with a subclass transition showed a significantly enrichment of the module “skyblue1”, while non-transitioning tumors have higher protein abundance in module “coral1”. **b** Most abundant proteins for tumor with a subclass transition (referring to module “skyblue1”). **c** Most abundant proteins for tumor with a subclass transition (referring to module “skyblue1”). **d** Integrating public transcriptomic single-cell data showed an AC-/OPC- and MES-like character in tumors with a subclass transition. **e** Volcano plot of -log10 (*p* value) against log2 fold change representing the differently abundant proteins at time of diagnosis between tumors of with a subclass transition as compared to tumors without a transition. **f** Dot plot illustrating most significantly upregulated gene ontology terms at time of diagnosis in glioblastoma with a subclass transition. **e**–**g** Protein abundance of stem cell markers from tumor tissue of first surgery were compared between glioblastomas without transition and with mesenchymal transition.* AC* astrocytic,* OPC* oligodendrocyte precursor cell,* MES* mesenchymal,* SOX* Sex determining region Y-box 2,* PROM* prominin

To extend our analysis of transitioning and non-transitioning tumors, we employed an integrative analysis of paired epigenetic and tumor proteome (mass spectrometry) datasets of our glioblastoma samples. First, we computed a scale-free gene expression network (WGCNA) which revealed that the gene expression module “skyblue1” was significantly higher abundant in transitioning tumors while non-transitioning tumors were enriched for the module “coral1” (Fig. 4a). Further analysis of these modules demonstrated contrary expressed proteins between both subgroups (Fig. 4b, c). When projecting the proteome module of glioblastomas with a subclass transition (“*skyblue1*”) onto a public single-cell dataset, results showed an enrichment of AC-like cells and somewhat reduction of MES-like cells at time of diagnosis when compared to tumors without a subclass transition (Fig. 4d). These findings primarily reflect the signature of the *RTK* subclasses present in the newly diagnosed tumors, specifically the *RTK II* subclass [32]. In addition, analyzing proteomic differential abundances showed various proteins significantly upregulated in transitioning tumors (Fig. 4e). Results of a consecutive ontology analysis revealed an upregulation of several terms associated with metabolic and catabolic processes in newly diagnosed glioblastoma with a subclass transition (Fig. 4f). Altered metabolism has previously been shown to be a hallmark of high-grade gliomas and its prognostic relevance in glioblastoma has recently been demonstrated [16, 42]. However, the association between a methylation-based subclass transition in recurrent glioblastoma and metabolic processes is as yet uncharacterized. Since a link between altered tumor metabolism and receptor tyrosine kinase signaling and their associated gene alterations has been described [4], we analyzed copy number profiles of all samples (Supplementary Table 1). Genomic alterations inferred from the methylation data at first surgery showed amplification of *EGFR* (50.0%) and loss of *CDKN2A/B* (53.1%) as the most frequent alterations. However, none of the genomic alterations were correlated with a subclass transition (Supplementary Table 1). Lastly, further characterization of the enriched stem cell-like state compartment in glioblastomas with a mesenchymal transition revealed an increased abundance of stem cell markers *SOX2*, *PROM-1*, and *Nestin* (Fig. 4e–g).

### Subclass transition did not influence patients outcome

**Fig. 5:**
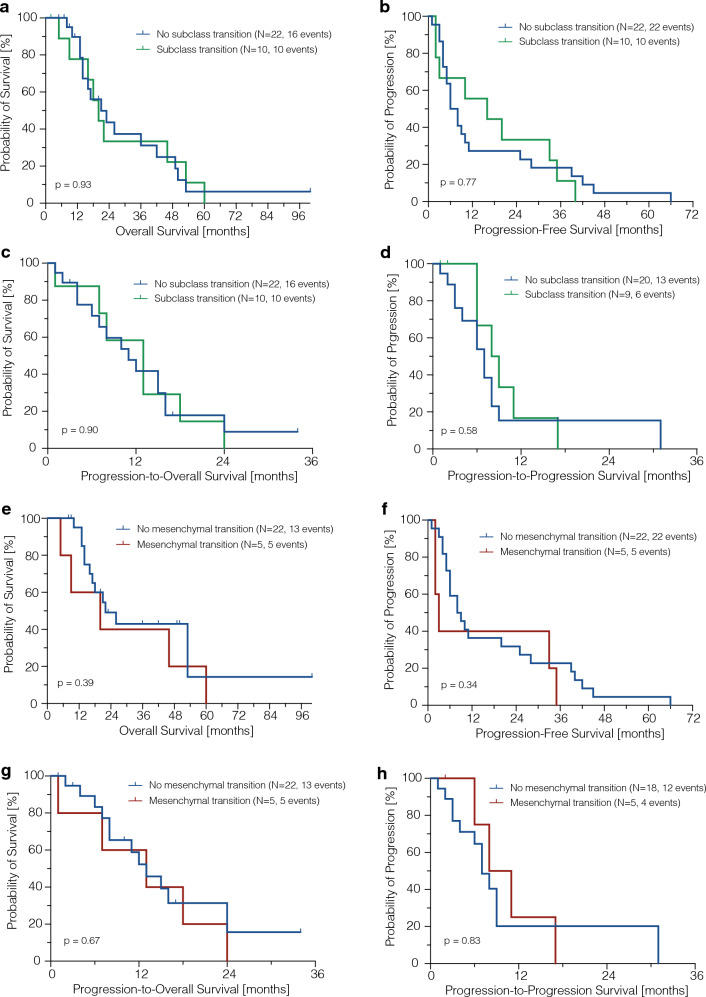
Kaplan Meier curves representing the survival outcome regarding DNA methylation subclass transition. **a**–**d** Survival outcome between patients with a methylation subclass change and without a subclass change, and **e**–**h** dependent on a potential mesenchymal subclass change

We further explored whether a change in DNA methylation subclass had an impact on patient survival, conducting an outcome analysis on patients from the Hamburg institution (*n* = 32). With a median (SD) follow-up of 19.5 (5.9) months, 26 (81.3%) deaths were observed. Clinical characteristics, including age, adjuvant treatment, and Karnofsky score at the time of initial and recurrent surgery, were comparable between the two groups (Table 1). Notably, patients without a subclass change had a higher rate of GTR at the first surgery (*p* = 0.04). Survival analysis revealed comparable overall survival (OS) (*p* = 0.93, Fig. 5a) and progression-free survival (PFS) (*p* = 0.77, Fig. 5b). After recurrent surgery, outcomes remained comparable between the two groups when analyzing postoperative survival (POS) (*p* = 0.90, Fig. 5c) and progression-free postoperative survival (PPS) (*p* = 0.58, Fig. 5d). Furthermore, no significant survival differences were observed in patients undergoing a mesenchymal transition (Fig. 5e–h).

## Discussion

A factor contributing to the aggressive behavior of glioblastoma is the spatiotemporal heterogeneity, which poses a major challenge in finding an optimal therapeutic regimen and targeting tumor cells [37, 52, 57]. Although the spatial heterogeneity of DNA methylation subclasses in newly diagnosed glioblastoma has been described previously [53, 59], their robustness and its clinical significance during disease progression remains of great interest. Our study provides the following important findings: (1) Temporal changes in DNA methylation subclasses were observed in 28.2% of tumors, with the majority of transitions occurring towards the mesenchymal subclass. (2) Transition to the dominant DNA methylation subclass was more frequent after incomplete tumor resection; however, there was no association with adjuvant treatment modalities or the time between initial and recurrent surgery. (3) Glioblastomas with subsequent mesenchymal transition demonstrated a higher stem cell-like state but a decreased immune cell state, as well as upregulated metabolic and catabolic processes at the time of diagnosis. (4) Despite a decreased immune cell state in tissue, significantly higher circulating immune signatures, as well as the cytokines IL-6 and IL-18, were observed in serum at the time of diagnosis in patients with a mesenchymal transition. (5) Conversely, matched tumor tissue at recurrence showed an increased immune cell state but decreased stem cell-like state after a mesenchymal transition, revealing an opposite cell composition than at the initial diagnosis. (6) The survival outcome of glioblastoma patients with a subclass transition was comparable to that of patients without such a transition.

Tumor profiling based on DNA methylation facilitates more accurate differentiation of brain tumor subgroups and allows subclassification of glioblastoma, with *RTK I*, *RTK II*, and MES being the most common subclasses [6, 49]. For these DNA methylation subclasses, two studies investigated the spatial heterogeneity of malignant gliomas [53, 59]. Wenger and colleagues analyzed 38 biopsies from 12 patients with newly diagnosed glioblastoma and reported intratumoral heterogeneity of the dominant DNA methylation subclass in 5 patients, demonstrating the existence of different DNA methylation subclasses within one tumor [59]. A more recent study by Verburg et al. failed to confirm this degree of heterogeneity and reported more stable DNA methylation subclasses across tumors when analyzing 133 biopsies from 16 patients, 7 of whom diagnosed with glioblastoma [53]. While these studies investigated spatial differences of DNA methylation subclasses, we focused on possible temporal heterogeneity and found that the dominant DNA methylation subclass changed in 28.2% of 39 tumors between first and recurrent surgery, with the transition to the mesenchymal subclass being most likely. These findings indicate that DNA methylation subclasses exhibit greater stability compared to transcriptional subtypes, since large-scale studies investigating the transcriptional glioblastoma subtypes reported about a transition of the dominant subtype in about 50.0% of the patients [26, 52]. Varn and colleagues observed a most frequent switch to the transcriptional mesenchymal subtype which is in accordance with our findings [52]. In the past years, the mesenchymal transition was investigated extensively and various drivers, such as radiation, and immune cell interactions were identified [14, 17, 32, 43]. When investigating potential surgery- or treatment-related factors contributing to a DNA methylation subclass transition, we identified incomplete removal of the contrast-enhancing tumor. In contrast, treatment characteristics and time between surgeries as well as genetic alterations had no impact. To obtain a more comprehensive understanding of the epigenetic profile, we conducted an analysis of CpG sites in newly diagnosed glioblastoma. Through this analysis, we identified specific methylation signatures that were differentially abundant and associated with metabolic and catabolic processes in glioblastoma cases exhibiting a subclass transition. While the link between DNA methylation subclass heterogeneity and increased tumor metabolism remains unclear, changes in metabolic profiles have been identified as a hallmark of high-grade gliomas [16] and have demonstrated prognostic impact in glioblastoma patients recently [42]. An underlying mechanism could be the interaction with stem cells, as demonstrated previously [45], which is also related to another finding of our study. Integrating cell state composition analysis showed a markedly increased stem cell-like state in tumors with mesenchymal transition at time of diagnosis, highlighting the importance of stem cells in transition processes and progress [15, 52, 57]. This result is consistent with our finding that increased residual contrast enhancement after first surgery favors subclass transition since peripheral, contrast-enhanced tumor areas are considered as one niche of stem cells [33]. However, at time of recurrence, there was a comparatively reduced stem cell percentage, which agrees with the results of Varn et al. [52].

The interplay between stem cells and immune cells in the tumor environment has been illustrated several times [15, 31, 46]. Here, we found a decreased immune state in newly diagnosed glioblastoma with mesenchymal transition, in opposition to the stem cell state, which is inverted in recurrent tumor tissue after mesenchymal transition. Interestingly, signatures of increased circulating immune cells in the serum of patients with a mesenchymal transition were already observed at the time of diagnosis, so that here a stem cell-associated immune evasion preceding mesenchymal transition during the course of the disease can be hypothesized and could be subject of future studies. The relevance of the change in immune cell composition observed here in tumors with a mesenchymal transition is consistent with two recent studies [10, 56]. Furthermore, our study identified possible soluble factors which are differentially concentrated in patients serum at time of diagnosis. The cytokines IL-6 and IL-18 were highly elevated in serum of mesenchymal-transitioning tumors. This might be explained with an interplay with stem cells given the existing literature [2, 9, 21, 55], and for both factors it appears reasonable to consider these as biomarkers in further studies with larger cohorts.

Since recurrent glioblastoma and especially the mesenchymal transcriptional subtype are considered particularly aggressive, we asked whether a DNA methylation subtype transition is relevant to patient survival [14]. Previously, studies demonstrated worse PFS and OS in patients with a transition to the mesenchymal transcriptional subtype at recurrence [26, 57]. This might be explained in conjunction to our study with the increased stem cell-like state at time of diagnosis in these transitioning tumors, as already demonstrated in recurrent gliomas [52].

However, in our study, survival of patients with and without a subclass transition was comparable. The lack of prognostic relevance of methylation subclass transition is most plausibly consistent with previous studies that reported comparable survival between *RTK I*, *RTK II*, and MES tumors at the time of initial diagnosis [11, 13, 25, 60]. Although methylation subclass does not appear to be a general prognostic marker, its clinical importance in predicting the probability of glioblastoma-associated seizure [12, 38] and the benefit of surgical resection [13] has been previously highlighted. This underscores the need for additional rapid [1, 19] and intraoperative [54] methods to detect the methylation subclass.

## Conclusion

In summary, our study unveiled that 28.2% of glioblastoma cases manifested a transition in the dominant DNA methylation subclass, predominantly clustering towards the mesenchymal subclass. This mesenchymal transition was accompanied by significant changes in stem cell-like and immune-like components, both at the time of diagnosis and recurrence. These findings underscore the importance of considering such transitions in the development of future targeted therapies for recurrent tumors.

### Supplementary Information

Below is the link to the electronic supplementary material.

Supplementary file1 a) Visualization of DNA tumor purity between “matching” and “non-matching” recurrent samples by using the DKFZ classifier v12.8. ns=non-significant. b) Visualization of DNA input amount between “matching” and “non-matching” recurrent samples by using the DKFZ classifier v12.8. ns=non-significant (PDF 404 kb)

Supplementary file2 Genetic alterations analyzed from DNA methylation data from tumor tissue at time of diagnosis (PDF 32 kb)

## Data Availability

All data and idat files are available from the corresponding author upon reasonable request.
