# Supplementary figures and images for: Temporal change of DNA methylation subclasses between matched newly diagnosed and recurrent glioblastoma
Source: Acta Neuropathol. 2024 Jan 20;147(1):21. doi: 10.1007/s00401-023-02677-8 (PMC10799798; doi:10.1007/s00401-023-02677-8)

Supplementary Figure 1

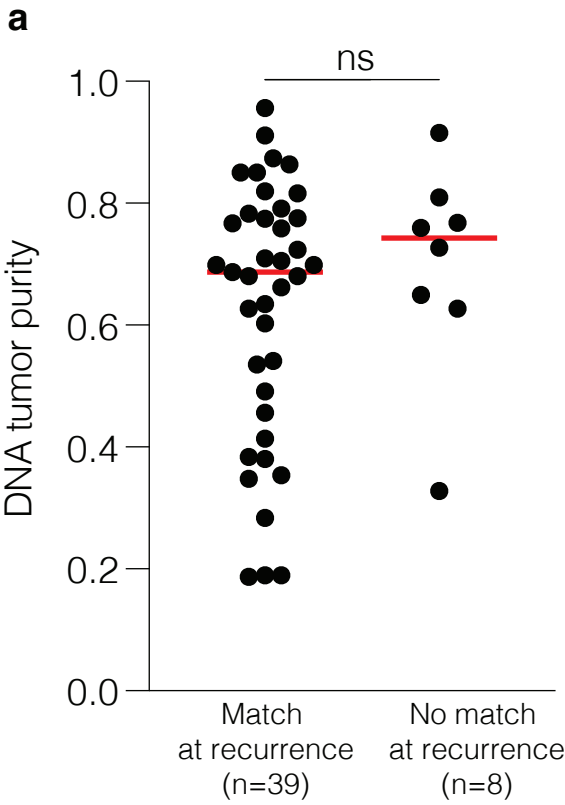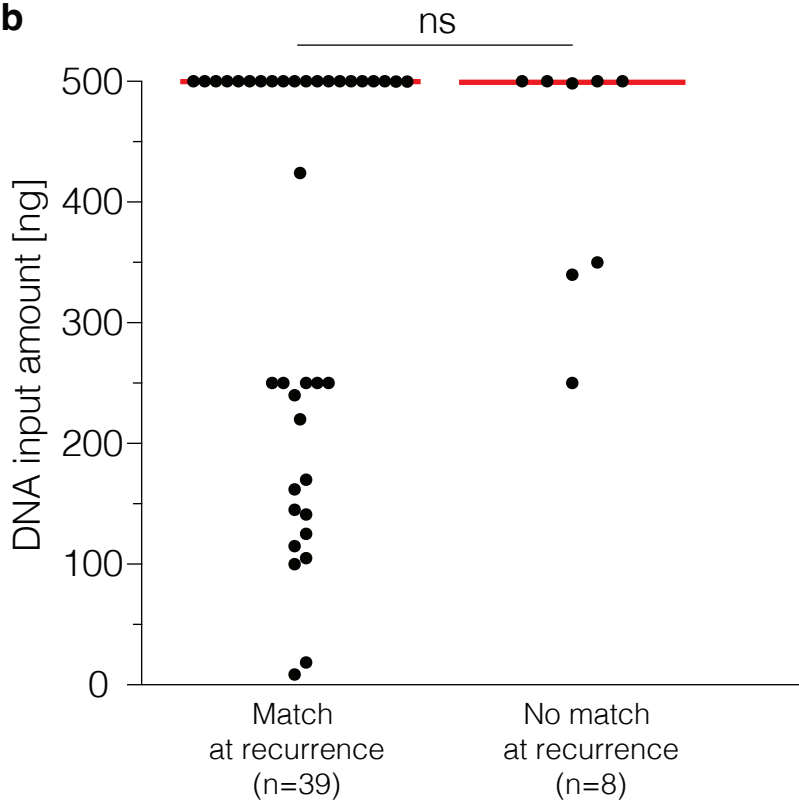

Supplement: Supplementary file 1 — Supplementary file1 a) Visualization of DNA tumor purity between “matching” and “non-matching” recurrent samples by using the DKFZ classifier v12.8. ns=non-significant. b) Visualization of DNA input amount between “matching” and “non-matching” recurrent samples by using the DKFZ classifier v12.8. ns=non-significant (PDF 404 kb) [file 401_2023_2677_MOESM1_ESM.pdf]
