# Supplementary material for: Temporal change of DNA methylation subclasses between matched newly diagnosed and recurrent glioblastoma
Source: Acta Neuropathol. 2024 Jan 20;147(1):21. doi: 10.1007/s00401-023-02677-8 (PMC10799798; doi:10.1007/s00401-023-02677-8)
Supplement: Supplementary file 2 — Supplementary file2 Genetic alterations analyzed from DNA methylation data from tumor tissue at time of diagnosis (PDF 32 kb) [file 401_2023_2677_MOESM2_ESM.pdf]

Supplementary table 1

Gene	N	No subclass transition (n=22)	Subclass transition (n=10)	P value
MDM4, n (%)				
No alteration	30 (93.8)	21 (95.5)	9 (90.0)	0.56
Amplification	2 (6.2)	1 (4.5)	1 (10.0)	
FGFR3, n (%)				
No alteration	30 (93.8)	22 (100.0)	8 (80.0)	0.09
Amplification	2 (6.2)	0 (0.0)	2 (20.0)	
PDGFR, n (%)				
No alteration	29 (90.6)	19 (86.4)	10 (100.0)	0.22
Loss	3 (9.4)	3 (13.6)	0 (0.0)	
TERT, n (%)				
No alteration	28 (87.5)	20 (90.9)	8 (0.0)	0.46
Loss	4 (12.5)	2 (9.1)	2 (20.0)	
EGFR, n (%)				
No alteration	16 (50.0)	11 (50.0)	5 (50.0)	0.99
Amplification	16 (50.0)	11 (50.0)	5 (50.0)	
MET, n (%)				
No alteration	30 (93.8)	21 (95.5)	9 (90.0)	0.56
Amplification	2 (6.2)	1 (4.5)	1 (10.0)	
CDKN2A/B, n (%)				
No alteration	15 (46.9)	11 (50.0)	4 (40.0)	0.59
Loss	17 (53.1)	11 (50.0)	6 (60.0)	
CDK4, n (%)				
No alteration	28 (87.5)	19 (86.4)	9 (90.0)	0.77
Amplification	4 (12.5)	3 (13.6)	1 (10.0)	
MDM2, n (%)				
No alteration	30 (93.8)	21 (95.5)	9 (90.0)	0.56
Amplification	2 (6.3)	1 (4.5)	1 (10.0)	
C19MC, n (%)				
No alteration	26 (81.3)	18 (81.8)	8 (80.0)	0.90
Loss	6 (18.8)	4 (18.2)	2 (20.0)	
